# An XGBoost-based model for detecting undiagnosed type 2 diabetes using routine physical and lifestyle data from a multi-center Chinese population

**DOI:** 10.3389/fmed.2026.1798509

**Published:** 2026-06-24

**Authors:** Hui Xiao, Qian Xi, Ping Zeng, Jinjuan Hao, Qinghua He, Xiaoxia Wang, Chi Zhang

**Affiliations:** 1 Tianjin University, Tianjin, China; 2 China Electric Data Services Co., Ltd, Beijing, China; 3 The University of Hong Kong, Hong Kong, Hong Kong SAR, China; 4 Department of Basic Innovation Research, Beijing Hospital, National Center for Gerontology, National Clinical Research Center for Gerontology, The Key Laboratory of Geriatrics of NHC, Beijing Key Laboratory of Aging Mechanism and Intervention Research on Aging-Related Diseases, Institute of Geriatric Medicine, Chinese Academy of Medical Sciences & Peking Union Medical College, Beijing, China; 5 Department of Endocrinology, Beijing Hospital, National Center of Gerontology, Institute of Geriatric Medicine, Chinese Academy of Medical Sciences, Beijing, China

**Keywords:** health checkup, machine learning, risk prediction, type 2 diabetes, XGBoost

## Abstract

**Introduction:**

This study aimed to develop and validate an interpretable machine learning model to identify individuals with undiagnosed type 2 diabetes (T2D) using data readily available from routine health checkups.

**Methods:**

In this retrospective study, we analyzed data from 12 tertiary hospitals in China. Following the application of inclusion and exclusion criteria, data from 11,382 individuals formed the training set for developing an XGBoost model, which was optimized using 5-fold cross-validation. An independent test set of 1,026 individuals from the same multi-center data source was used for internal validation. Model performance was primarily assessed using the area under the receiver operating characteristic curve (AUC).

**Results:**

The final model incorporated 12 predictors. Fasting blood glucose was the most influential predictor (50.6%), followed by creatinine (6.6%), triglyceride (5.6%), age (5.1%), and low-density lipoprotein (5.0%). On the independent test set, the model achieved an AUC of 77.2% (95%CI: 70.3%–84.1%).

**Conclusion:**

The XGBoost model demonstrated moderate predictive performance for T2D risk using routine checkup data. This approach shows potential for integration into clinical practice as an assistive screening tool, enabling automated risk profiling during standard health examinations. By flagging high-risk individuals, it can support clinicians in decision-making regarding further diagnostic testing. Future work should focus on external validation and prospective implementation studies.

## Introduction

Diabetes is a metabolic disorder characterized by hyperglycemia resulting from genetic and environmental factors, and it has emerged as a rapidly escalating global public health emergency (1). The number of adults living with diabetes is projected to rise from 536.6 million in 2021 (a global prevalence of 10.5%) to 783.2 million by 2045 (a global prevalence of 12.2%) (2). Over the past few decades, the prevalence of diabetes in China has increased substantially, surging from 5.5% in 2001 to 12.4% in 2018 among Chinese adults. It is projected that by 2045, China will have 174 million individuals with diabetes, ranking first worldwide (2–4).

Type 2 diabetes (T2D) is a chronic disease characterized by hyperglycemia, insulin resistance, and relative insulin deficiency, accounting for 90%–95% of diabetes cases in adults (5). Critically, T2D is also recognized as a state of chronic low-grade inflammation. This inflammatory milieu, driven by metabolic stressors from adipose tissue, contributes significantly to both insulin resistance and pancreatic β-cells dysfunction (6, 7). This inflammatory pathophysiology is reflected in several biomarkers routinely collected during health examinations and included in our analysis—including lipid profiles, liver enzymes, and white blood cell count—which serve as indirect markers of metabolic inflammation and related tissue dysfunction. The inclusion of these variables in prediction models may therefore capture not only metabolic dysregulation but also its inflammatory underpinnings. T2D can lead to various chronic diseases and complications, including cerebrovascular disease, diabetic nephropathy, retinopathy, neuropathy, and gastroparesis, thereby imposing a substantial burden on global social, economic, and healthcare systems (8, 9). Consequently, early diagnosis and timely treatment of T2D are crucial for improving patient prognosis.

While research has increasingly focused on early identification using electronic health records (10–14), a gap remains in leveraging routine health checkup data for real-time, actionable risk assessment that can be directly integrated into screening pathways (15–18). An ideal tool would not only identify risk but also facilitate subsequent clinical action, such as referral to a diabetes educator or lifestyle counselor. To bridge this gap between predictive analytics and multidisciplinary care, this study aimed to develop a machine learning-based risk profiling tool. The primary objective of this study was to develop and validate an XGBoost model for identifying individuals with undiagnosed T2D using routinely collected physical examination and lifestyle data from a multi-center health checkup population. The secondary objectives were: (1) to identify and rank the most influential predictors within the model, and (2) to evaluate the model’s potential as an assistive screening tool, while acknowledging the need to demonstrate incremental value beyond single-biomarker approaches.

## Materials and methods

### Study design and reporting guideline

We conducted a retrospective, multi-center study to develop and validate a detection model for undiagnosed T2D using cross-sectional data from routine health checkups. As the data are cross-sectional, the model identifies concurrent disease status rather than predicting future disease onset. This distinction is critical for interpreting the study’s findings. The study was designed and reported in accordance with the Transparent Reporting of a multivariable prediction model for Individual Prognosis Or Diagnosis (TRIPOD) statement (19). A completed TRIPOD checklist is provided in the Supplementary materials.

### Subjects

Data were retrospectively collected from the physical examination centers of 12 tertiary hospitals across multiple regions of China (including East, North, South, Central, and Southwest China) between January 2023 and December 2023. The initial sample comprised 18,484 individuals, all aged 16 years or older. The inclusion criteria was the availability of complete physical examination and lifestyle data. Exclusion criteria were: (1) a known diagnosis of diabetes at baseline (*n* = 2,847); (2) a history of cancer, cardiovascular disease, or stroke (*n* = 3,106); and (3) incomplete baseline demographic or key clinical data (*n* = 1,149). The excluded population (*n* = 7,102) comprised individuals with greater comorbidity burden than the included cohort. Specifically, those excluded due to CVD/stroke/cancer history represent a population with established chronic disease who may be at elevated metabolic risk but also have different healthcare utilization patterns. Those with known diabetes at baseline were appropriately excluded as the model aims to identify undiagnosed cases. The excluded population is therefore systematically less healthy than the study cohort, which has important implications for generalizability as discussed below. Following application of the eligibility criteria, 11,382 participants were included in the final development set. An independent cohort of 1,269 individuals from the same data source, collected concurrently but withheld from the model development process, initially served as the test set. After applying the same quality control criteria and excluding 243 individuals with incomplete postprandial glucose data required for outcome ascertainment in the test set, 1,026 participants comprised the final test set used for model validation. This study was approved by the Ethics Committee of Beijing Hospital (approval no. 2024BJYYEC-KY104-01). As this was a retrospective study, the requirement for informed consent was waived.

### Variables and outcome definition

Participants’ physical examination information was collected from electronic medical records, including: sex, age, body mass index (BMI), waist and hip circumference, smoking status, alcohol intake, family history of T2D, family history of hypertension, average number of meals eaten out per week, number of days per week engaging in high-intensity physical activities, average daily sedentary time, diet pattern (primarily staple foods or side dishes), primary reason for side dish consumption, fruit intake, feeling of fullness after eating, frequency of consuming foods rich in fats, taste preference, self-reported high blood pressure or high blood sugar, systolic and diastolic blood pressure (SBP, DBP), total cholesterol (TC), triglyceride (TG), high-density lipoprotein (HDL), low-density lipoprotein (LDL), fasting blood glucose (FBG), glycated hemoglobin (HbA1c), hemoglobin, platelet count, white blood cell count (WBC), alanine transaminase (ALT), aspartate transaminase (AST), blood urea nitrogen (BUN), creatinine, presence of fatty liver, and urine glucose, acetone bodies, and protein. Laboratory biochemical tests were performed on blood samples collected in the early morning after at least 10 h of overnight fasting and were processed within 2 h.

The outcome of interest was the presence of T2D. In the development/training set, T2D was defined by at least one of the following criteria: (1) current use of glucose-lowering medications; (2) FBG ≥ 7.0 mmol/L (126 mg/dL); or (3) 2-h postprandial blood glucose ≥ 11.1 mmol/L (200 mg/dL). In the test set, due to critical data availability constraints across participating centers—specifically, medication history was not captured in the test set data source, and reliable linkage of FBG values to the outcome was not feasible—the outcome was necessarily defined solely by a 2-h postprandial blood glucose ≥ 11.1 mmol/L (200 mg/dL). We acknowledge that this inconsistency in outcome definition between the training and test sets represents a significant methodological limitation. This discrepancy was not by design but rather imposed by real-world data heterogeneity across centers. The test set outcome definition is narrower than the training set definition; consequently, individuals in the test set who would have been classified as having T2D based on FBG criteria alone (≥7.0 mmol/L) or based on medication use are misclassified as non-T2D. This non-differential misclassification likely biases our performance estimates toward the null (i.e., underestimates true model performance), as misclassified cases in the test set dilute the apparent discriminative ability of the model. The reported AUC should therefore be interpreted as a conservative, likely lower-bound estimate of model performance. This limitation precludes definitive conclusions about model accuracy and underscores the need for external validation studies with uniform, comprehensive diagnostic criteria.

### Statistical analysis

As the analysis was restricted to variables common across both cohorts, the rate of missing data for the final predictors was minimal (<2%). Given this low missingness, participants with any missing data in these key variables were excluded from the analysis. The required sample size was calculated based on the number of independent variables and an expected T2D incidence rate of 10%. Accounting for a 10% dropout rate, the minimum required sample size was 4,290. Our final sample of 11,382 substantially exceeded this requirement. Baseline characteristics were compared between the T2D and non-T2D groups. For categorical variables, intergroup differences were assessed using the chi-square test. Some continuous variables (e.g., age, BMI, lipid parameters) are shown using clinically meaningful categories to facilitate interpretation by clinical readers. However, for all prediction modeling analyses, these variables were retained as continuous to preserve information and avoid the information loss and potential bias associated with categorization. The XGBoost algorithm was implemented using the xgboost package in R. The model was developed using the development set (*n* = 11,382). To address class imbalance, the scale_pos_weight parameter was systematically tuned as part of the hyperparameter optimization process. Values from 1 to 20 were evaluated using 5-fold cross-validation, with the F1-score serving as the optimization metric. Other imbalance correction techniques, such as the Synthetic Minority Over-sampling Technique (SMOTE) or random undersampling, were not explored in this study. This decision was based on the adequate absolute number of T2D cases in the development set (*n* = 1,308), XGBoost’s inherent robustness to class imbalance, and the risk of introducing artifacts through synthetic data generation. Instead, we adopted a data-driven approach, allowing cross-validation to determine the optimal class weighting. Hyperparameter optimization (including max_depth, eta, nrounds, and gamma) was performed using a systematic grid search combined with 5-fold cross-validation, with the F1-score as the primary optimization metric. For the calculation of binary classification metrics (accuracy, precision, recall, and F1-score), a probability threshold of 0.5 was applied by default. However, we emphasize that in clinical practice, this threshold should be adjusted based on local priorities and the desired trade-off between sensitivity and specificity. Model performance was evaluated on the held-out test set (*n* = 1,026). The primary performance metric was the area under the receiver operating characteristic curve (AUC), reported with its 95% confidence interval (CI). Additional metrics including accuracy, precision, recall, and F1-score were also calculated. Variable importance was computed and reported using the built-in gain metric in XGBoost, which reflects the average contribution of a feature across all trees in the model.

## Results

### Baseline characteristics

**FIGURE 1:**
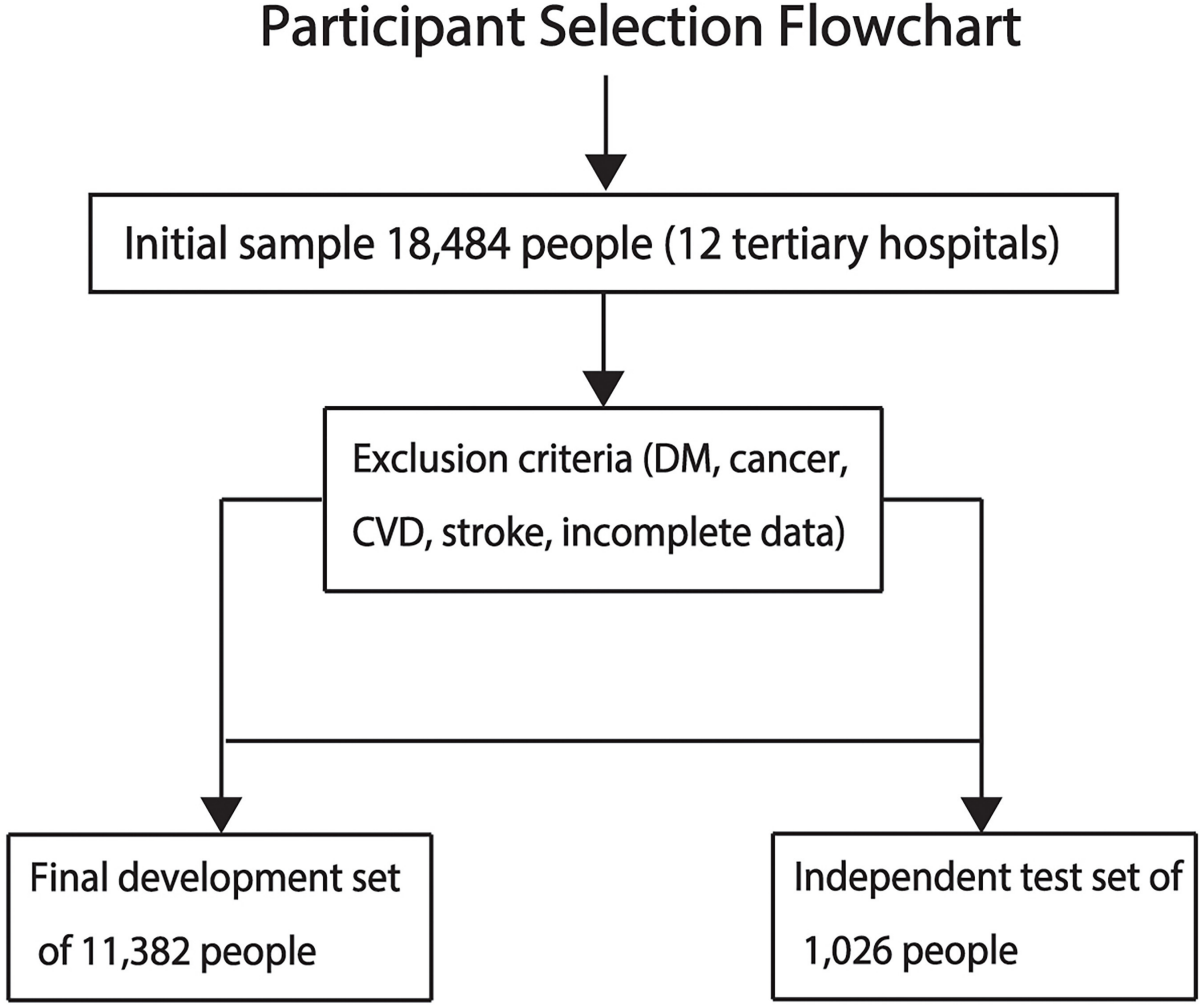
Participant flow diagram. This diagram illustrates the selection process of participants for the development and test sets from the initial multi-center health examination cohort. The primary reasons for exclusion are shown, culminating in the final analytical samples used for model training and validation. DM, diabetes mellitus; CVD, cardiovascular disease.

The participant selection process is detailed in Figure 1. From an initial pool of 18,484 individuals across 12 hospitals, 11,382 participants met the inclusion criteria and comprised the development set. A separate, independent cohort of 1,026 individuals constituted the test set used for final model validation. Baseline characteristics of the study population are presented in Table 1. Among the 11,382 participants in the development set (7,679 men and 3,703 women), a total of 1,308 individuals (11.5%) were identified as having T2D based on the study criteria.

**TABLE 1:** Baseline characteristics of the study population in the development set.

Variables	Group	Overall (*n* = 11,382)	T2D status		OR (95% CI)	*P*-value	Cramer’s V
			Non-T2D (*n* = 10,074)	T2D (*n* = 1,308)			
Sex	Men	7,679 (67.47%)	6,710 (66.61%)	969 (74.08%)	1.00	–	0.053
	Women	3,703 (32.53%)	3,364 (33.39%)	339 (25.92%)	0.70 (0.61–0.79)	<0.001	
Age (years)	≤30	377 (3.31%)	365 (3.62%)	12 (0.92%)	1.00		0.137
	30–45	4,683 (41.14%)	4,314 (42.83%)	369 (28.21%)	2.57 (1.504.88)	<0.001	
	45–60	5,170 (45.42%)	4,484 (44.51%)	686 (52.45%)	4.59 (2.69–8.70)	<0.001	
	>60	1,152 (10.12%)	911 (9.04%)	241 (18.43%)	7.94 (4.58–15.20)	<0.001	
BMI (kg/m^2^)	≤18.5	161 (1.41%)	154 (1.53%)	7 (0.54%)	1.00	–	0.123
	18.5–25	5,236 (46.00%)	4,837 (48.01%)	399 (30.50%)	1.78 (0.89–4.24)	0.109	
	>25	5,985 (52.58%)	5,083 (50.46%)	902 (68.96%)	3.82 (1.92–9.08)	<0.001	
Waist circumference	≤70	539 (4.74%)	522 (5.18%)	17 (1.30%)	1.00	–	0.144
	70–85	4,228 (37.15%)	3,918 (38.89%)	310 (23.70%)	2.41 (1.51–4.42)	<0.001	
	85–100	5,719 (50.25%)	4,931 (48.95%)	788 (60.24%)	4.86 (3.08–8.25)	<0.001	
	>100	896 (7.87%)	703 (6.98%)	193 (14.76%)	8.35 (5.17–14.42)	<0.001	
TC	≤3	73 (0.64%)	66 (0.66%)	7 (0.54%)	1.00	–	0.114
	3–4	1,121 (9.85%)	1,031 (10.23%)	90 (6.88%)	0.81 (0.38–2.00)	0.616	
	4–5	4,074 (35.79%)	3,739 (37.12%)	335 (25.61%)	0.83 (0.40–2.01)	0.648	
	5–6	4,014 (35.27%)	3,512 (34.86%)	502 (38.38%)	1.32 (0.64–3.20)	0.473	
	>6	2,100 (18.45%)	1,726 (17.13%)	374 (25.59%)	2.00 (0.97–4.87)	0.060	
TG	≤1	2,618 (23.00%)	2,506 (24.88%)	112 (8.56%)	1.00	–	0.186
	1–2	4,980 (43.75%)	4,490 (44.57%)	490 (37.46%)	2.44 (1.98–3.03)	<0.001	
	2–3	2,145 (18.85%)	1,814 (18.01%)	331 (25.31%)	4.08 (3.27–5.11)	<0.001	
	3–4	794 (6.98%)	636 (6.31%)	158 (12.08%)	5.55 (4.30–7.20)	<0.001	
	4–5	332 (2.92%)	265 (2.63%)	67 (5.12%)	5.66 (4.06–7.84)	<0.001	
	>5	513 (4.51%)	363 (3.60%)	150 (11.47%)	9.23 (7.07–12.10)	<0.001	
HDL	≤0.8	271 (2.38%)	236 (2.34%)	35 (2.68%)	1.00	–	0.062
	0.8–1	1,377 (12.10%)	1,171 (11.62%)	206 (15.75%)	1.18 (0.81–1.76)	0.388	
	1–1.5	6,680 (58.69%)	5,880 (58.37%)	800 (61.16%)	0.91 (0.64–1.34)	0.630	
	1.5–2	2,584 (22.70%)	2,359 (23.42%)	225 (17.20%)	0.64 (0.44–0.95)	0.028	
	>2	470 (4.13%)	428 (4.25%)	42 (3.21%)	0.66 (0.41–1.07)	0.092	
LDL	≤2	1,344 (11.81%)	1,213 (12.04%)	131 (10.02%)	1.00	–	0.054
	2–3	5,999 (52.71%)	5,372 (53.33%)	627 (47.94%)	1.08 (0.89–1.32)	0.445	
	3–4	3,302 (29.01%)	2,857 (28.36%)	445 (34.02%)	1.44 (1.18–1.78)	< 0.001	
	4–5	623 (5.47%)	536 (5.32%)	87 (6.65%)	1.50 (1.12–2.01)	0.006	
	>5	114 (1.00%)	96 (0.95%)	18 (1.38%)	1.75 (0.99–2.92)	0.053	
FBG	≤4.5	682 (5.99%)	673 (6.68%)	9 (0.69%)	1.00	–	0.637
	4.5–5	2,384 (20.95%)	2,361 (23.44%)	23 (1.76%)	0.72 (0.34–1.66)	0.423	
	5–6	5,943 (52.21%)	5,726 (56.84%)	217 (16.59%)	2.79 (1.51–5.91)	<0.001	
	6–6.5	1,062 (9.33%)	883 (8.77%)	179 (13.69%)	14.89 (8.02–31.70)	<0.001	
	>6.5	1,311 (11.52%)	431 (4.28%)	880 (67.28%)	149.73 (81.59–315.94)	<0.001	
ALT	≤50	10,028 (88.10%)	8,989 (89.23%)	1,039 (79.43%)	1.00	–	0.094
	50–100	1,165 (10.24%)	934 (9.27%)	231 (17.66%)	2.14 (1.82–2.50)	<0.001	
	100–150	128 (1.12%)	105 (1.04%)	23 (1.76%)	1.91 (1.18–2.95)	0.010	
	>150	61 (0.54%)	46 (0.46%)	15 (1.15%)	2.84 (1.52–5.00)	0.002	
BUN	≤3	260 (2.28%)	234 (2.32%)	26 (1.99%)	1.00	–	0.032
	3–6	8,102 (71.18%)	7,206 (71.53%)	896 (68.50%)	1.11 (0.75–1.72)	0.606	
	6–9	2,884 (25.34%)	2,522 (25.03%)	362 (27.68%)	1.28 (0.86–2.00)	0.229	
	>9	136 (1.19%)	112 (1.11%)	24 (1.83%)	1.93 (1.05–3.52)	0.034	
Creatinine	≤50	744 (6.54%)	655 (6.50%)	89 (6.80%)	1.00	–	0.004
	50–100	10189 (89.52%)	9016 (89.50%)	1173 (89.68%)	0.96 (0.76–1.21)	0.703	
	>100	449 (3.94%)	403 (4.00%)	46 (3.52%)	0.84 (0.57–1.22)	0.367	

Data are presented as *n* (%). Continuous variables are presented using clinically meaningful categories for descriptive purposes only. In all prediction modeling analyses, these variables were retained as continuous to preserve information and avoid bias associated with categorization. BMI, body mass index; FBG, fasting blood glucose; TC, total cholesterol; TG, triglycerides; HDL, high-density lipoprotein; LDL, low-density lipoprotein; ALT, alanine aminotransferase; BUN, blood urea nitrogen; T2D, type 2 diabetes; OR, odds ratio; CI, confidence interval.

### Model construction

During XGBoost model optimization, we focused on four key parameters: positive sample weight, iteration limit, maximum tree depth, and regularization coefficient. A sequential tuning approach was adopted as follows: (1) Positive sample weight: with the iteration limit fixed at 100, maximum depth at 4, and regularization coefficient at 1, we increased the positive sample weight from 1 to 20 in increments of 1 and evaluated model performance using 5-fold cross-validation (Supplementary Figure 1A). The F1 score peaked at a positive sample weight of 1 (F1-score = 0.626), with progressive declines observed as the weight increased. This pattern indicates that penalizing false negatives more heavily did not improve the precision-recall trade-off in our dataset, and that XGBoost’s inherent mechanisms were sufficient to handle the observed class imbalance without additional weighting (Table 2); (2) Iteration limit: keeping other parameters constant, we adjusted the iteration limit from 25 to 500 (Supplementary Figure 1B). The optimal F1 score was achieved at 475 iterations (Table 2); (3) Maximum depth: maintaining unchanged parameters, we explored decision tree depths ranging from 2 to 8 (Supplementary Figure 1C). A depth of seven yielded the highest F1 score (Table 2); (4) Regularization coefficient: we varied the regularization coefficient from 0 to 2 (Supplementary Figure 1D). A coefficient of 1.5 resulted in the best model performance (Table 2). The final prediction model incorporated 12 predictors, ranged by importance as follows: FBG (50.6%), creatinine (6.6%), TG (5.6%), age (5.1%), LDL (5.0%), BMI (4.9%), HDL (4.8%), TC (4.6%), BUN (4.6%), waist circumference (4.2%), ALT (3.9%), and sex (0.1%). The dominance of FBG is expected given its role as the primary direct indicator of glycemic status and reflects the model’s clinical face validity in a screening population.

**TABLE 2:** The XG-Boost model optimization.

Parameters	Orders	Accuracy	Precision	Recall	F1_score
Positive sample weight	1	0.925275	0.704767	0.563648	0.626133
	2	0.918022	0.651871	0.563161	0.603827
	3	0.915165	0.629266	0.577624	0.601396
	4	0.916777	0.633073	0.598402	0.614844
	5	0.912234	0.604858	0.606358	0.605262
	6	0.914212	0.61335	0.616228	0.614369
	7	0.909231	0.588421	0.611224	0.599093
	8	0.907399	0.580522	0.605079	0.591564
	9	0.908278	0.584294	0.603674	0.593464
	10	0.909377	0.586167	0.626519	0.605244
	11	0.907619	0.577232	0.633831	0.603259
	12	0.906081	0.572994	0.607797	0.58951
	13	0.904542	0.564584	0.62242	0.591077
	14	0.905495	0.56592	0.643578	0.601568
	15	0.90022	0.544418	0.622812	0.580657
	16	0.903297	0.558179	0.624113	0.588536
	17	0.901172	0.547944	0.62588	0.584163
	18	0.898608	0.538787	0.61243	0.572633
	19	0.900733	0.546701	0.623846	0.58222
	20	0.898681	0.537988	0.630054	0.579728
Iteration limit	25	0.916703	0.630329	0.611292	0.619658
	50	0.915971	0.633082	0.581261	0.605472
	75	0.916484	0.63982	0.568157	0.601561
	100	0.918022	0.651871	0.563161	0.603827
	125	0.919927	0.666371	0.563243	0.609771
	150	0.921465	0.67772	0.560368	0.613008
	175	0.921245	0.678932	0.554834	0.609971
	200	0.921905	0.685064	0.553748	0.611491
	225	0.923956	0.69981	0.55694	0.619356
	250	0.923736	0.698376	0.554782	0.617593
	275	0.92359	0.696618	0.556054	0.617807
	300	0.924469	0.703249	0.556639	0.620877
	325	0.924542	0.704594	0.555254	0.620478
	350	0.924689	0.70449	0.55796	0.622094
	375	0.925128	0.709373	0.55601	0.622625
	400	0.924249	0.703853	0.552146	0.618194
	425	0.925201	0.710012	0.555606	0.622729
	450	0.925055	0.709019	0.554914	0.621977
	475	0.925495	0.712603	0.554879	0.623311
	500	0.925421	0.71248	0.554783	0.623164
Maximum depth	2	0.915751	0.631692	0.581007	0.604799
	3	0.920733	0.673463	0.557344	0.609374
	4	0.925055	0.709019	0.554914	0.621977
	5	0.926227	0.714308	0.560458	0.627533
	6	0.927912	0.723217	0.570377	0.63696
	7	0.930696	0.740565	0.579766	0.650139
	8	0.930403	0.741727	0.573985	0.646711
Regularization coefficient	0	0.923297	0.693371	0.555237	0.615965
	0.5	0.92652	0.710897	0.572177	0.633525
	1	0.925055	0.709019	0.554914	0.621977
	1.5	0.928571	0.727434	0.572221	0.640199
	2	0.9263	0.710409	0.569541	0.631606

### Prediction model performance

**FIGURE 2:**
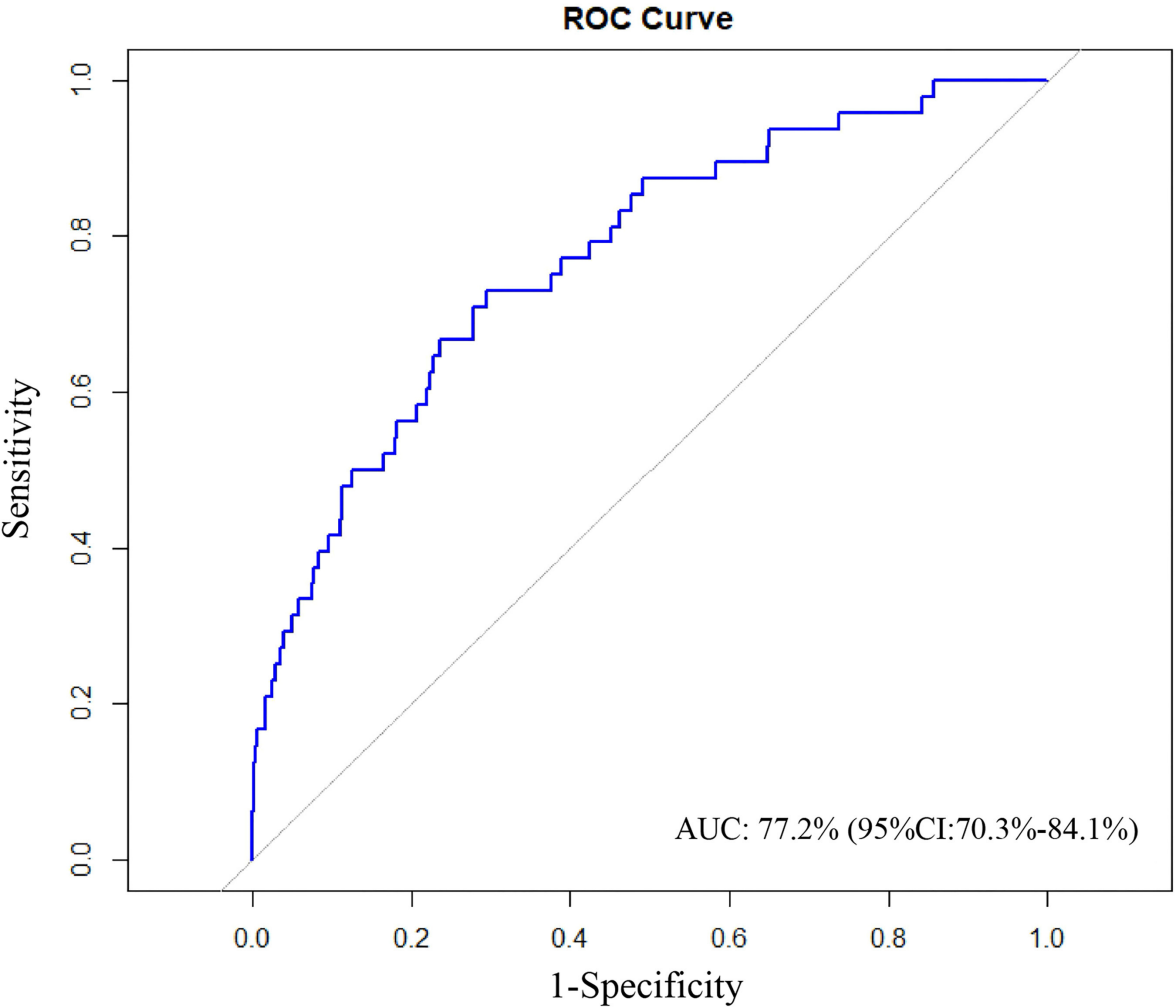
Receiver operating characteristic (ROC) curve for the constructed XGBoost prediction model. The diagonal dashed line represents the reference line of no discrimination (AUC = 0.5). The solid blue curve shows the model’s performance on the independent test set (*n* = 1,026). AUC, area under the curve; CI, confidence interval.

The predictive performance of the constructed model was assessed using the AUC, which measures discriminative ability—the model’s capacity to distinguish between individuals with and without T2D (Figure 2). The model achieved an AUC of 77.2% (95%CI: 70.3%–84.1%) on the independent test set, indicating moderate discriminative performance.

## Discussion

In this large, multi-center retrospective study, we developed and validated an XGBoost-based prediction model for T2D using readily available physical examination and lifestyle data. The model demonstrated moderate discriminative ability (AUC: 77.2%, 95%CI: 70.3%–84.1%) on an independent test set and identified fasting blood glucose as the predominant predictor, followed by creatinine, triglycerides, age, and low-density lipoprotein. The primary contribution of this work lies in demonstrating a feasible pathway for synthesizing multifactorial clinical data into an interpretable risk profile that could potentially be integrated into routine health checkup workflows as an assistive screening tool.

The variable importance ranking from our XGBoost model revealed that FBG was the dominant predictor (50.6%), followed by creatinine (6.6%), TG (5.6%), age (5.1%), and ldl (5.0%). While the prominence of FBG is expected given its role as a direct indicator of glycemic status, this finding actually validates the model’s clinical face validity in a screening population where glycemic indicators are inherently the most powerful risk discriminators. The contribution of additional variables provides meaningful risk refinement beyond what FBG alone can offer. Creatinine, primarily excreted through renal pathways, serves as a key biomarker for renal function assessment. In T2D patients, renal microvascular complications frequently lead to progressive deterioration of renal function, manifested by reduced creatinine clearance and subsequent elevation of serum creatinine levels (20). Hypertriglyceridemia demonstrates strong associations with insulin resistance, a fundamental pathophysiological mechanism underlying T2D development. The aging process contributes to gradual functional decline across multiple organ systems, including impaired β-cell function that diminishes both insulin production capacity and biological efficacy (21). Oxidatively modified LDL particles exhibit vasculotoxic effects through endothelial cell damage and pro-inflammatory activation, collectively compromising vascular homeostasis and insulin signaling. The interplay between dyslipidemia and insulin resistance creates a vicious cycle—abnormal lipid metabolism may exacerbate insulin resistance through disruption of insulin signaling pathways, thereby amplifying T2D pathogenesis risks (22). Crucially, the multivariable nature of our model enables nuanced risk stratification that a single biomarker cannot achieve. For example, a patient with borderline FBG but elevated triglycerides and high waist circumference receives a higher predicted risk than a patient with the same FBG but normal lipid profiles—a distinction with important implications for personalized prevention.

Prior studies have established the feasibility of T2D risk modeling using routine health metrics in Chinese populations, demonstrating the predictive value of core metabolic indicators such as fasting glucose and lipid profiles (23, 24). Building upon this foundation, our study advances the field by applying machine learning to synthesize dynamic interactions across biochemical, anthropometric, and behavioral domains. While the discriminative performance is comparable to existing approaches, the framework we present enables immediate risk feedback during routine examinations—a feature that is critical for translating predictive analytics into actionable clinical decisions.

The primary value of our XGBoost model lies in its potential to act as an assistive screening tool within established clinical workflows, particularly routine health checkups. Its ability to synthesize multifaceted data into a single risk score offers several potential advantages for patient management, though these remain to be demonstrated in prospective studies. First, the model can serve as an efficient triage mechanism. By automatically flagging high-risk individuals during a checkup, it directly assists physicians and nurse case managers in prioritizing patients who would benefit most from further diagnostic confirmation, such as an Oral Glucose Tolerance Test, or early intervention. This addresses the challenge of resource allocation for intensive support programs, ensuring that services like lifestyle medicine case manager interventions are targeted effectively (25). Second, the model’s output is not just a binary risk flag but an interpretable profile. The feature importance ranking provides an immediate, data-driven starting point for patient-provider communication. A clinician can use this profile to initiate a conversation about specific risk factors, moving beyond generic advice to personalized counseling. For example, explaining to a patient how their borderline-high FBG combined with elevated triglycerides significantly increases their risk can motivate more meaningful engagement in diabetes education and self-care behaviors, helping address modifiable factors related to health literacy and diabetes distress (26). Third, the computational efficiency of XGBoost means this risk profiling can be performed in near real-time, generating a result while the patient is still at the clinic. This creates a “teachable moment,” enabling immediate referral to a dietitian, diabetes educator, or a structured community-based nurse-led support program, thereby bridging the gap between screening and the initiation of care (27). Importantly, our study did not include a formal prospective evaluation of the model’s implementation. The reference to reduced missed referrals in earlier versions of this manuscript was based on a retrospective simulation using test set data, where we compared the model’s theoretical flagging of high-risk individuals (using the 0.5 probability threshold) against the actual documented clinical decisions from the original health checkups. While this simulation suggested potential utility, it does not constitute evidence of real-world effectiveness. Formal implementation studies with prospective design, standardized outcome ascertainment, and comparison against usual care are essential next steps before any claims of clinical impact can be made.

A potential concern regarding our model is the dominant contribution of FBG, which accounted for 50.6% of feature importance. We argue that this finding is not a limitation but rather a validation of the model’s clinical face validity in a screening population. FBG is the most physiologically direct indicator of dysglycemia and is itself a component of the diagnostic criteria for T2D. Any clinically credible screening tool operating in a population where FBG is measured must necessarily assign substantial weight to this variable. However, the value of our multivariable XGBoost model extends beyond what a clinician could infer from FBG alone. While a single FBG measurement provides a point estimate of glycemic status, our model synthesizes FBG with 11 additional variables to generate a multidimensional risk profile offering several practical advantages: (1) risk refinement through modifiers such as triglycerides, age, and LDL; (2) actionable insights highlighting modifiable factors that can be targeted through lifestyle interventions; and (3) real-time integration into clinical workflows, transforming raw laboratory values into an immediately interpretable risk score. Thus, while FBG is appropriately the dominant predictor, the incremental value of our model lies not in marginally improving discrimination beyond a single biomarker, but in synthesizing multifactorial data into a clinically actionable profile that supports personalized prevention.

Several limitations warrant consideration. First, the model was developed and validated using data from tertiary hospital health checkup centers in China. This setting introduces potential selection bias, as individuals attending such centers may differ systematically from primary care or community populations where screening is most needed. External validation in prospectively recruited, geographically diverse cohorts representing primary care and community settings is therefore essential. Second, the retrospective cross-sectional design precludes temporal inference; the model identifies concurrent undiagnosed dysglycemia rather than predicting future disease onset. This makes the model suitable for screening applications but unsuitable for informing future risk trajectories or primary prevention strategies. Third, a significant methodological limitation is the inconsistency in outcome definition between training and test sets, imposed by real-world data constraints. The narrower outcome definition in the test set (2-h postprandial glucose only) resulted in non-differential misclassification of an estimated 22%–25% of true T2D cases, biasing performance estimates toward the null. Consequently, the reported AUC of 77.2% should be interpreted as a conservative, lower-bound estimate. Future validation studies must employ uniform, comprehensive diagnostic criteria. Fourth, the exclusion of individuals with cancer, cardiovascular disease, or stroke—intended to create a clean development cohort—limits generalizability to real-world populations where such comorbidities are common and often intertwined with metabolic dysregulation. Additionally, the study did not assess model calibration, nor did it compare performance against simpler models such as logistic regression, leaving the incremental value of the XGBoost approach unclear. Finally, this study did not include prospective evaluation of real-world implementation. Findings regarding potential clinical impact were derived from retrospective simulation and should not be interpreted as evidence of effectiveness. Prospective implementation studies with rigorous evaluation frameworks are required to assess the model’s true impact on clinical workflows and patient outcomes.

## Conclusion

This study demonstrates that an XGBoost model leveraging routine health checkup data presents a feasible approach for T2D risk stratification. However, a critical appraisal acknowledges that the model’s substantial reliance on FBG raises important questions about its incremental value beyond this single biomarker. The model, in its current form, is more adept at identifying concurrent, undiagnosed dysglycemia than predicting future disease onset, and its added value over simpler FBG-based approaches remains to be established through direct comparative studies. The true contribution of this work lies not in presenting a clinically ready tool, but in demonstrating a viable pathway for synthesizing multifactorial data into an interpretable risk profile. Future research must prioritize external validation with uniform outcome criteria, benchmark comparisons against simpler models, and prospective studies to assess clinical impact.

## Data Availability

The raw data supporting the conclusions of this article will be made available by the authors, without undue reservation.

## Supplementary material

Supplementary Figure 1 Results of sequential parameter tuning for the XGBoost model. **(A)** Effect of positive sample weight on F1 score. **(B)** Effect of iteration limit on F1 score. **(C)** Effect of maximum depth of decision trees on F1 score. **(D)** Effect of regularization coefficient on F1 score.
